# Description of the new HIV-1 intersubtype B/C circulating recombinant form (CRF146_BC) detected in Brazil

**DOI:** 10.1590/0074-02760230214

**Published:** 2024-09-23

**Authors:** Rodrigo Cunha Oliveira, Darren Martin, Juliana Sacramento Mota de Souza, Luiz Carlos Júnior Alcântara, Monick Lindenmeyer Guimarães, Carlos Brites, Joana Paixão Monteiro-Cunha

**Affiliations:** 1 Universidade Federal da Bahia, Departamento de Bioquímica e Biofísica, Núcleo de Bioinformática, Salvador, BA, Brasil; 2 University of Cape Town, Institute of Infectious Disease and Molecular Medicine, Department of Integrative Biomedical Sciences, Computational Biology Group, Cape Town, South Africa; 3 Fundação Oswaldo Cruz-Fiocruz, Instituto Oswaldo Cruz, Laboratório de Flavivírus, Rio de Janeiro, RJ, Brasil; 4 Fundação Oswaldo Cruz-Fiocruz, Instituto Oswaldo Cruz, Laboratório de AIDS e Imunologia Molecular, Rio de Janeiro, RJ, Brasil; 5 Universidade Federal da Bahia, Complexo Hospitalar Prof Edgard Santos, Laboratório de Pesquisa em Infectologia, Salvador, BA, Brasil

**Keywords:** CRF146_BC, HIV-1, recombination, Brazil

## Abstract

**BACKGROUND:**

The human immunodeficiency virus 1 (HIV-1) infections in Brazil are predominantly caused by two subtypes, B and C.

**OBJECTIVES:**

Here we present the characterisation of a novel HIV-1 recombinant form, indicating a new Brazilian CRF_BC, named CRF146_BC.

**METHODS:**

RDP, JphMM and Simplot recombination tools were used to evaluate the mosaic pattern.

**FINDINGS:**

In this work, we identified three HIV-1 nucleotide sequences previously classified as unique recombinant forms (URFs), plus one new partial genome sharing the same BC recombination pattern. The mosaic genome is almost entirely represented by the subtype C sequence, with a small subtype B recombination region in the pol gene, at the Integrase level. The phylogenetic analyses strongly indicate a common origin between the strains, which were isolated in Rio Grande do Sul, Rio de Janeiro and Bahia states.

**MAIN CONCLUSIONS:**

Thus, the new HIV-1 CRF146_BC is circulating in three different Brazilian regions: South, Southeast and Northeast.

The human immunodeficiency virus (HIV) discovered in the early 1980s is classified into two types: HIV-1 and HIV-2.^1^ ^,^ ^2^ HIV-1 is highly diversified, being represented by four groups (M, N, O and P). The M group is further classified into 10 subtypes (A, B, C, D, F, G, H, J, K and L), sub-subtypes (A1-A8 and F1, F2) and circulating or unique recombinant forms (URF).^2^ ^,^ ^3^ *In vivo* co-infection or super-infection with different strains can result in the emergence of hybrid viruses.^2^ ^,^ ^4^ ^,^ ^5^ These viruses are generated through the genic recombination mechanism, which occurs mainly during retrotranscription, from pure or recombinant parental strains, resulting in recombinant forms.^5^ When recombinant viruses sharing an identical mosaic structure are isolated from three or more epidemiologically unrelated individuals, they are designated circulating recombinant forms (CRF).^2^ To define a new CRF, three near-full length genomes (NFLG) or two NFLGs in conjunction with a partial sequence of a third strain are sufficient. Recombinant forms that do not meet these criteria are designated as URF.^2^ ^,^ ^5^

The subtype C accounts for nearly one-half of HIV-1 infections worldwide (46.6%), whereas the B subtype is globally distributed.^4^ Recombinant strains are widely spread and estimated to be responsible for nearly 23% of infections worldwide.^4^ Numerous URFs and 157 CRFs have been described over the years (https://www.hiv.lanl.gov/components/sequence/HIV/crfdb/crfs.comp). Among these, 14 are CRF_BC, 11 of which were identified in China,^6^ but some were also identified in Brazil and Italy.^7^ ^,^ ^8^ The first (CRF07_BC) was described in 2000^5^ and the last (CRF118_BC) in 2021.^9^

In Brazil, the subtype B is the prevalent HIV-1 genotype, followed by the C and F1 subtypes, together with distinct recombinant forms (BF1 and BC mainly).^10^ In this country, B and C subtypes cause 81% of the HIV-1 infections and a BC CRF (CRF31_BC), in addition to several BC URF have been described.^7^ ^,^ ^11^ Here we present the characterisation of a novel HIV-1 recombinant form, indicating a new Brazilian CRF_BC, named CRF146_BC.

## SUBJECTS AND METHODS

Sample 223 presented here (accession number OR260538) was collected in 2019 from an HIV-1 infected patient followed at Professor Edgard Santos University Hospital in Salvador (Bahia), a capital city in the Northeast region of Brazil. Both Hospital and the Federal University of Bahia Ethics Committee approved the study (Ethics Committee Number: 2.831.993). The participant supplied written informed consent for specimen collection and subsequent analyses. The nested-polymerase chain reaction (PCR) and DNA sequencing protocol were applied as previously described,^12^ using the specific HIV-1 primers, covering four overlapping regions. However, only genomic fragments corresponding to *integrase/vif* and *nef* genes (positions 4172 to 5198 and 8694 to 9512 relative to HXB2 reference genome, respectively) were successfully obtained, while other genomic regions did not amplify. RDP,^13^ JphMM^14^ and Simplot^15^ recombination tools were used to evaluate the mosaic pattern. The Simplot analysis were conducted based in the follow parameters: 350 nucleotides sliding window and steps of 30 bases, in accordance with Passaes et al. and Pessôa et al.^16^ ^,^ ^17^ A breakpoint between C and B subtypes was found in the *integrase/vif* region (positions 4837+-77 - 5071+-21 HXB2 coordinates), while the *nef* gene region reveals a pure C fragment.

A search for complete genome sequences of BC recombinants has been performed in the Los Alamos National Laboratory (LANL) database (https://www.hiv.lanl.gov/components/sequence/HIV/search/search.html), using the following criteria: complete genome; subtypes B + C; include recombinants. Then, a manual search for sequences identified as pure BC with more than 7000 nt in length was conducted using the BioEdit program V.7.2.5.^18^ As a result, 225 NFLG BC sequences were obtained. In order to investigate and compare the recombination patterns, sequences were analysed using the aforementioned recombination tools. Out of the 225 BC recombinants found in the database, 16 NFLG sequences were isolated in Brazil. Of these, three samples, previously described as URF_BC, were identified with similar breakpoints in the *integrase/vif* genes and classified as pure subtype C in the *nef* gene: GQ365652 isolated in Rio Grande do Sul, KT427778 and KT427790, both isolated in Rio de Janeiro. In order to investigate the phylogenetic relationships, these four samples (OR260538, GQ365652, KT427778 and KT427790) were aligned with 134 recombinant sequences representing 14 CRF_BC, plus 42 pure subtype reference sequences collected from LANL database. The alignment was performed using MAFFT online program^19^ (http://mafft.cbrc.jp) under the command: mafft --thread 8 --threadtb 5 --threadit 0 --reorder -- auto input > output and manually edited using BioEdit software.^18^ The maximum likelihood (ML) trees were reconstructed using IQ-TREE 1.6.8 webserver^20^ (Command: path_to_iqtree -s aln.fasta -m TEST -bb 1000 -alrt 1000) and RDP5^13^ The reliability of each cluster was evaluated by analysing 1,000 bootstrap (BS) replicates and with the approximate likelihood ratio test (aLRT) based on the Shimodaira-Hasegawa-like procedure.^21^ The ML trees were visualised with the FigTree program version 1.4.4 (http://tree.bio.ed.ac.uk/software/figtree/). In order to confirm the recombination, the alignment was fragmented at the breakpoints and the segments (subtype C concatenated fragment - *integrase/vif* and *nef* - and subtype B *integrase* fragment) were submitted to ML analysis as described above.

## RESULTS

**Fig. 1:**
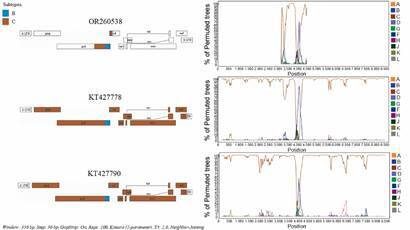
recombination pattern of human immunodeficiency virus 1 (HIV-1) CRF146_BC strains. CRF146_BC breakpoints were detected using the jumping profile hidden Markov model (JpHMM) and simplot tools. Simplot analysis was performed using a sliding window of 350 nucleotides and steps of 30 bases

Comparing sample OR260538 BC recombinant fragment with the other three NFLG sequences from Brazil [Fig. 1, Supplementary data (Fig. 1)], we identified similar breakpoints in: GQ365652 (4834+-75 - 5085+-21), KT427778 (4831+-80 - 5080+-19) and KT427790 (4851+-75 - 5085+-21). However, sample GQ365652 presented two other small subtype B fragments in the reverse transcriptase region (2986-3089 and 4172-4315). The detection of these subtype B insertions could only be detected by informative site analysis, since bootscanning analysis of these samples, only showed a discrete decrease in the similarity with subtype C (data not shown).^16^

**Fig. 2:**
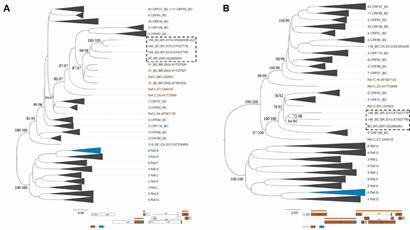
maximum likelihood (ML) analyses showing the phylogenetic relationships of the Brazilian CRF146_BC recombinant viruses with other human immunodeficiency virus 1 (HIV-1) BC circulating recombination forms (CRFs) (n = 134) and pure subtype reference (n = 42) sequences. Sequences sharing CRF146_BC recombination pattern formed a monophyletic group shown within the box. Trees were rooted by the midpoint. The subtype/CRF classification and the number of sequences within each collapsed cluster are indicated. The statistical support is indicated only at key nodes as approximated likelihood ratio test (aLRT) and bootstrap (BS) values. Trees were built under the GTR+I+G evolutionary model and visualised in the Figtree v1.4.4 software. Horizontal branch lengths were drawn to scale with the bar at the bottom indicating the nucleotide substitutions per site. Trees were built based on (A) BC *integrase/vif* and C *nef* concatenated genomic fragments as in sample OR260538 (4172 - 5198 and 8694 - 9412 relative to HIV-1 reference strain (HXB2) and (B) near-full length genomes (NFLG) sequences without OR260538 (nucleotides 805 - 8912 relative to HXB2 reference strain). The genomic region used to build each tree is shown in the HIV-1 genomic map below the topology (orange line).

**Fig. 3:**
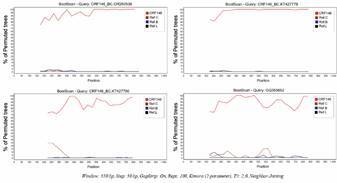
Bootscan analysis (SimPlot) showing the relationship between Brazilian CRF146 recombinant sequences. In this analysis, each CRF146 query (OR260538, KT427778, KT427790 and GQ365652) is more similar to the CRF146 group (containing the other three recombinant isolates) than to pure subtypes B and C reference sequences. Analyses were performed using the integrase genomic fragment, based on sample OR260538 sequence length.

**Fig. 4:**
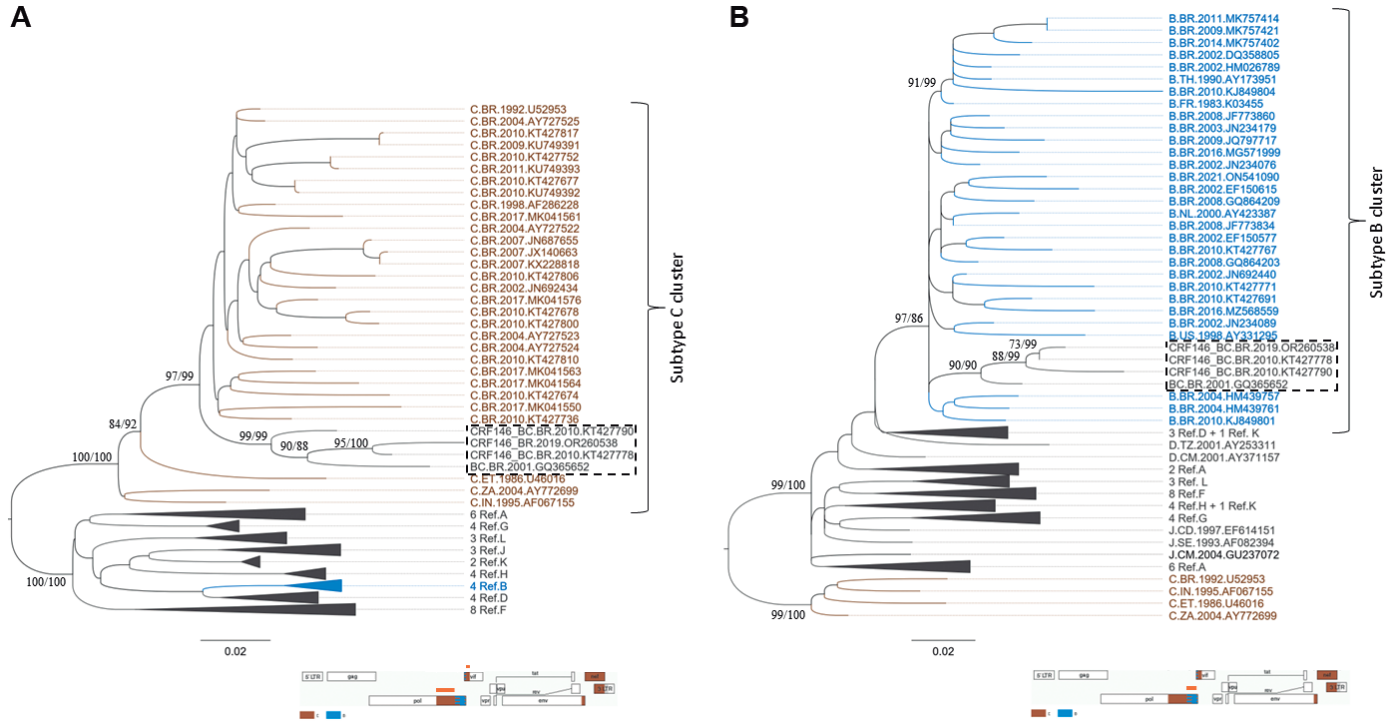
CRF146_BC recombinants viruses among Brazilian human immunodeficiency virus 1 (HIV-1) subtypes B and C samples. Sequences were fragmented at the breakpoints and the segments were submitted to maximum likelihood (ML) analysis with Los Alamos reference set. (A) C *integrase/vif* concatenated fragment (positions 4172 to 4834 and 5071 to 5190 relative to HIV-1 reference strain (HXB2,) and (B) B *integrase* fragment (nucleotides 4833 - 5066 relative to HXB2 reference strain. The statistical support is indicated only at key nodes as approximated likelihood ratio test (aLRT) and bootstrap values. Trees were built under the TIM2+I+G evolutionary model and visualised in the Figtree v1.4.4 software. Horizontal branch lengths were drawn to scale with the bar at the bottom indicating the nucleotide substitutions per site.

Next, these BC recombinant viruses were submitted to phylogenetic analyses with 134 HIV-1 nucleotide sequences of other BC CRFs and 42 pure subtypes, retrieved from LANL database.^20^ ML trees (Fig. 2) were reconstructed based on the fragment size of sample OR260538 (Fig. 2A) and NFLG sequences (Fig. 2B). Moreover, ML analyses of the individual fragments (subtype C concatenated fragment - *integrase/vif* and *nef* - and subtype B *integrase* fragment) using the appropriate evolutionary model confirmed the Bootscanning recombination results [Supplementary data (Fig. 2)]. Sequences OR260538, GQ365652, KT427778 and KT427790 grouped together with aLRT (approximated likelihood ratio test) > 90 and BS values > 94%. The ML trees generated in RDP5 software, showed similar topologies, reinforcing the close genetic relationship between these viruses and their branching apart of HIV-1 pure subtypes and other BC CRFs [Supplementary data (Fig. 3)]. Bootscanning analysis (Fig. 3) showed that BC recombinant sequences inside this monophyletic group were more similar to each other (OR260538, KT427778, KT427790 and GQ365652) than to reference sequences of pure HIV-1 subtypes. In addition, two other trees were generated using a panel of pure subtype B and C sequences from Brazil (Fig. 4). The four BC recombinant strains also grouped together as monophyletic clusters in both ML trees: inside the larger subtype C clade in the *integrase/vif* concatenated tree (Fig. 4A) and inside the subtype B clade in the smaller integrase fragment (Fig. 4B). These observations reinforce the straight phylogenetic relationships among these viral isolates.

## DISCUSSION

In Brazil, both subtype C and CRF31_BC were first identified in the Southern region and the subtype C have been spreading northward the country (Southeast, Central West, North and Northeast regions).^7^ ^,^ ^22^ This region accounts for 53,8% of HIV-1 subtype C infections in Brazil.^10^ Out of the four samples representing the new CRF, one (GQ365652) were sampled in Rio Grande do Sul (Southern region), two (KT427778 and KT427790) were from Rio de Janeiro State (Southeastern region) and one (OR260538) from Bahia State (Northeastern region). In addition, Passaes and collaborators,^16^ found the same breakpoint (4834+-75 - 5085+-21) in URF_BC sequences from Rio Grande do Sul State (Southern region) and suggested that second-generation recombination events, involving BC recombinant strains as parental, may be favouring the increase of the new genotypes, once both pure subtypes are prevalent in the country. In this regard, sample GQ365652 presented two additional subtype B fragments (< 200 bp) in *pol* gene,^16^ which were not observed in the other two NFLG sequences (KT427778 and KT427790). Since GQ365652 presented very close phylogenetic relationships with all other isolates in CRF146 group, grouping together in all six generated trees, we can speculate that these short (< 150nt) subtype B fragments could have been acquired throughout a second-generation recombination event.

In brief, here we described a novel CRF_BC in Brazil (CRF146_BC), which is circulating in, at least, three out of five geographic regions of the country. The ongoing emergence of new HIV-1 M genotypes emphasises the need to sustain genomic surveillance in Brazil. More specific studies will be necessary to determine whether, relative to its parentals this new CRF is either evolutionarily fitter or has altered pathogenicity.
