# Supplementary material for: Description of the new HIV-1 intersubtype B/C circulating recombinant form (CRF146_BC) detected in Brazil
Source: Mem Inst Oswaldo Cruz. 2024 Sep 23;119:e230214. doi: 10.1590/0074-02760230214 (PMC11421422; doi:10.1590/0074-02760230214)
Supplement: Supplementary file 1 [file 1678-8060-mioc-119-e230214-s.pdf]

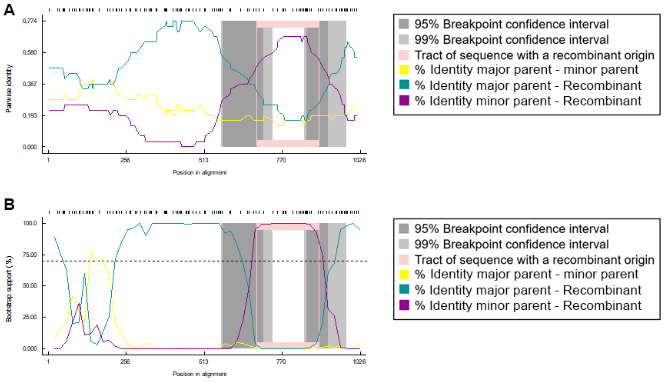

Fig. 1: recombination pattern of human immunodeficiency virus 1 (HIV-1) integrase fragment from sample 223 (OR260538) obtained through RDP (A) and Bootscan (B) methods, implemented in RDP5 tool. Yellow line: pairwise identity between major (Ref.C.BR.U52953) and minor (Ref.B.TH.AY173951) parents; green line: pairwise identity between major parent (Ref.C.BR.U52953) and the recombinant query (OR260538); purple line: pairwise identity between minor parent (Ref.B.TH.AY173951) and the recombinant query (OR260538). Dark gray: 95%Breakpoint confidence interval; light gray: 99% Breakpoint confidence interval (CI).

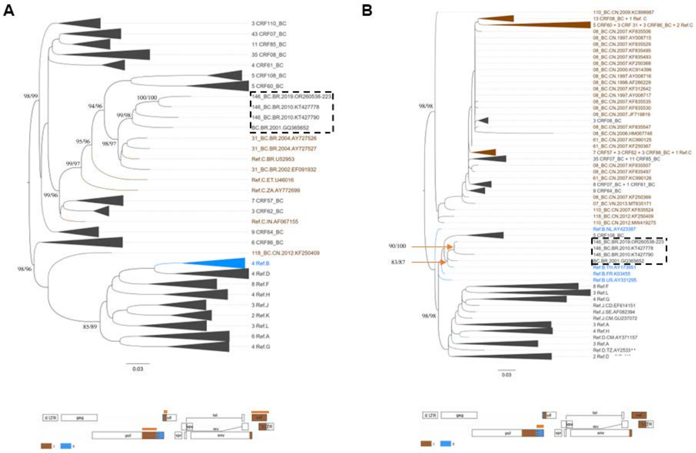

Fig. 2: maximum likelihood (ML) analyses showing the phylogenetic relationships of the Brazilian CRF146_BC recombinant viruses with other human immunodeficiency virus 1 (HIV-1) BC circulating recombination forms (CRFs) (n = 134) and pure subtype reference (n = 42) sequences. Sequences sharing CRF146_BC recombination pattern formed a monophyletic group shown within the box. Trees were rooted by the midpoint. The subtype/CRF classification and the number of sequences within each collapsed cluster are indicated. The statistical support is indicated only at key nodes as approximated likelihood ratio test (aLRT) and bootstrap values. Trees were built under the GTR+I+G evolutionary model and visualised in the Figtree v1.4.4 software. Horizontal branch lengths were drawn to scale with the bar at the bottom indicating the nucleotide substitutions per site. Trees were built based on (A) subtype C concatenated fragment (*integrase/vif* and *nef*) and (B) B subtype *integrase* fragment; (nucleotides 4833 - 5066 relative to HXB2 reference strain). The genomic region used to build each tree is shown in the HIV-1 genomic map below the topology (orange line).

CC BY

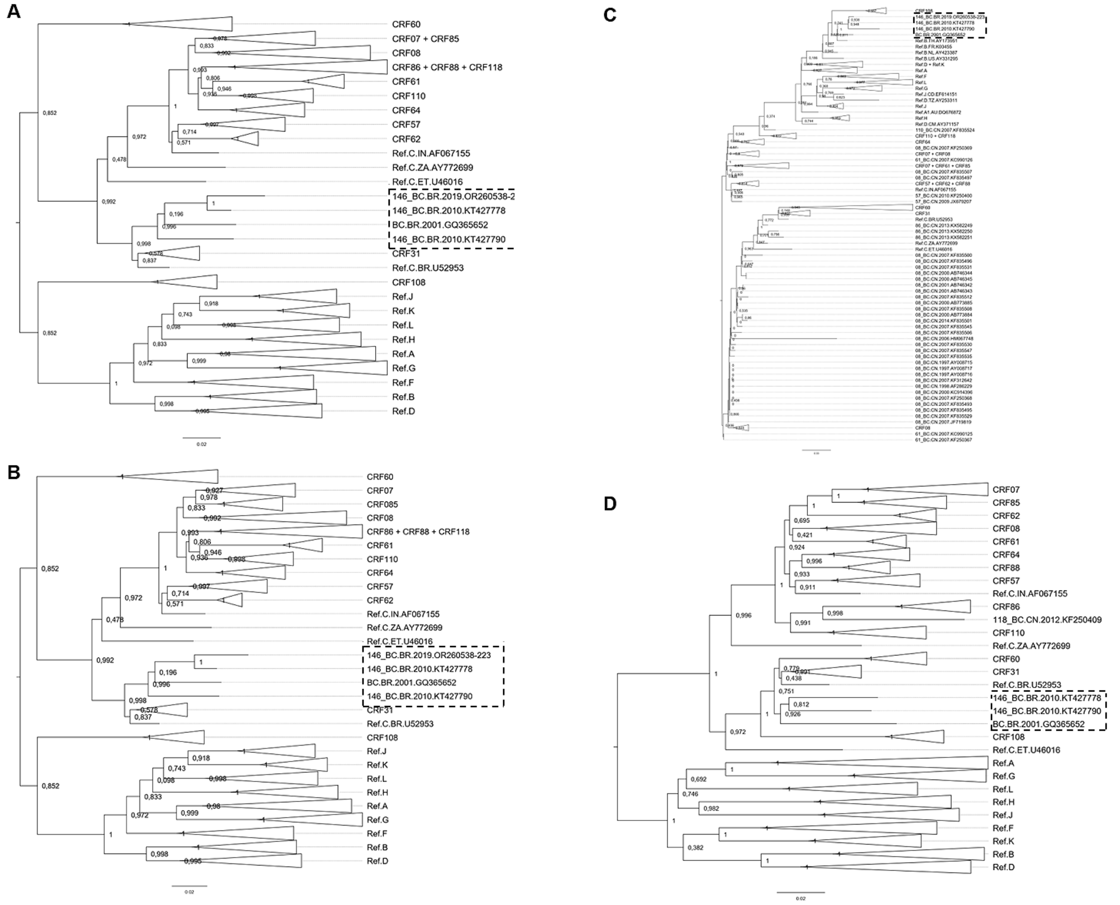

Fig. 3: RDP5 maximum likelihood (ML) analyses showing phylogenetic relationships of 180 human immunodeficiency virus 1 (HIV-1) sequences including new CRF146_BC, 134 sequences belonging to 14 circulating recombination forms (CRFs) and 42 subtype reference sequences. The trees were rooted by the midpoint and in the collapsed clusters. The subtype/CRF classification and the number of sequences within each collapsed cluster are indicated. The statistical support is bootstrap values. The trees were visualized in the Figtree v1.4.4 software. Horizontal branch lengths are drawn to scale with the bar at the bottom indicating the nucleotide substitutions per site. (A) BC *integrase* and C *nef* concatenated fragments; (B) subtype C fragment (*integrase* and *nef*) concatenated (C) B subtype *integrase* fragment; (nucleotides 4833 -5066 relative to HXB2 reference strain) and (D) near-full length genomes (NFLG) sequences without OR260538 (nucleotides 805 -8912 relative to HXB2 reference strain).
